# Cost-Effectiveness of Internet of Things–Based Management of Home Noninvasive Positive Pressure Ventilation in Patients With Chronic Obstructive Pulmonary Disease and Hypercapnic Chronic Respiratory Failure: Trial-Based Economic Evaluation

**DOI:** 10.2196/71340

**Published:** 2026-05-14

**Authors:** Ruihua Feng, Jiu Cheng, Yueying Cui, Xi Wang, Yinghao Lv, Weipeng Jiang, Chenjun Zhou, Yuanlin Song, Hui Liu

**Affiliations:** 1 Institute of Medical Information, Chinese Academy of Medical Sciences & Peking Union Medical College, Yabao Road 3, Beijing, 100020, China, 86 010-52328605; 2 Union Hospital, Tongji Medical College, Huazhong University of Science and Technology, Wuhan, Hubei, China; 3 Shenzhen Health Development Research and Data Management Center, Guangdong, China; 4 Department of Pulmonary Medicine and Critical Care Medicine, Zhongshan Hospital, Fudan University, Shanghai, China; 5 Department of Pulmonary Medicine, Zhongshan Hospital, Fudan University, Shanghai, China; 6 School of Biomedical Engineering, Tsinghua University, Beijing, China; 7 Shanghai Respiratory Research Institute, Fudan University, Shanghai, China

**Keywords:** chronic obstructive pulmonary disease, noninvasive positive pressure ventilation, hypercapnic chronic respiratory failure, Internet of Things, telemedicine, cost-effectiveness analysis, economic evaluation

## Abstract

**Background:**

The management of chronic obstructive pulmonary disease (COPD) places a significant burden on health care systems worldwide. Home noninvasive positive pressure ventilation (NPPV) is an established treatment option associated with significant benefits for patients with COPD and hypercapnic chronic respiratory failure. Internet of Things (IoT)–based management may improve communication between patients and physicians and strengthen the integration and comprehensiveness of home NPPV telemonitoring. However, the economic value of such systems remains insufficiently understood.

**Objective:**

This study aimed to assess the cost-effectiveness of IoT-based management versus standard management of home NPPV in patients with COPD and hypercapnic chronic respiratory failure.

**Methods:**

A Markov decision analytic model was developed to simulate real-world COPD progression and predict health outcomes and costs associated with IoT-based and standard management of home NPPV. COPD progression consisted of 4 health states: stable period, nonserious exacerbation period, serious exacerbation period, and death. Efficacy and cost inputs were primarily sourced from a published multicenter, prospective, randomized controlled trial and supplemented by official Chinese databases where necessary. Quality-adjusted life years (QALYs) were used as effect indicators for this model, which were derived from COPD Assessment Test scores. The discounted lifetime cost per QALY gained was calculated from the Chinese health care payer perspective, and sensitivity analyses were conducted to test the robustness of model results across different assumptions.

**Results:**

Compared with standard NPPV, IoT-based NPPV increased costs by ¥3607.26 and improved QALYs by 0.24 per person across the lifetime horizon, resulting in an incremental cost-effectiveness ratio of ¥15,030.25 per QALY, with a 93.6% probability of being cost-effective at the given willingness-to-pay threshold (currency conversion to US dollars was based on the average exchange rate in 2019 [US $1=¥6.9]). Base case results were also robust to multiple one-way sensitivity analyses, with the main drivers being hospitalization costs for the standard and IoT-based NPPV groups during the serious exacerbation period.

**Conclusions:**

IoT-based NPPV was cost-effective compared with standard NPPV for patients with COPD and hypercapnic chronic respiratory failure.

## Introduction

Chronic obstructive pulmonary disease (COPD) is a chronic airway disease characterized by airflow obstruction, with a high rate of disability and mortality, and it is also one of the diseases that need to be prevented and controlled by the Healthy China Initiative (2019‐2030) [1]. COPD is the sixth leading driver of the global burden of disease and imposes a substantial economic burden [2]. From 1990 to 2023, COPD consistently ranked among the top three level 3 causes of age-standardized mortality attributable to noncommunicable diseases in China [3]. The annual per capita direct medical cost for patients with COPD in China accounted for 33.33% to 118.09% of the local average annual income [4], with hospitalization and home oxygen therapy being the main driving factors for direct costs [5]. It is estimated that COPD will cost the world economy Int $4.326 trillion from 2020 to 2050, with China and the United States facing the largest economic burden of COPD [6]. With population aging in China, the burden of COPD is still rising. The prevention and control of COPD remain challenging. Effective management of stable patients with COPD is essential to improve their quality of life and reduce medical costs. Therefore, seeking the most economical and effective treatment and management program for patients with COPD is imperative.

A recent meta-analysis confirmed that home noninvasive positive pressure ventilation (NPPV) in treating patients with hypercapnic COPD was significantly associated with a lower mortality risk, all-cause hospital admission, and intubation [7]. The 2023 report of the Global Initiative for Chronic Obstructive Lung Disease (GOLD) [8], the latest guidelines from the European Respiratory Society [9], and the American Thoracic Society [10] support the use of home NPPV for patients with stable hypercapnic COPD. Therefore, NPPV has become an established treatment for patients with stable hypercapnic COPD. Telemonitoring of home NPPV is an important tool for optimizing follow-up. Using the Internet of Things (IoT)–based medical platform may improve the integration and comprehensiveness of telemonitoring and management of home NPPV [11]. The IoT cloud platform collects information on adherence, ventilator parameters, and physiological indicators and provides daily reports and follow-up summaries to the health care team. Therefore, it could improve the exchange of information between patients and physicians who prescribed NPPV, and between health professionals and the external companies that provide ventilator services, deliver individualized and fully integrated care management, as well as respond quickly to patient needs. These factors are crucial for guaranteeing treatment adherence and effectiveness. Relevant studies also showed that IoT-based home NPPV management is emerging and has produced positive results [12,13].

Hospitalization costs are a major component of the total cost of COPD exacerbations [14]. The use of IoT may save costs by reducing avoidable hospitalizations [15-17]. These potential cost savings need to offset the increased costs associated with IoT services. Therefore, it is vital to investigate the influence of IoT on the overall use of health care resources and to evaluate its cost-effectiveness to determine whether it represents a good value for money. A small number of studies have confirmed that in patients with chronic respiratory failure caused by neuromuscular or thoracic diseases (eg, obstructive sleep apnea, amyotrophic lateral sclerosis, and barrel chest), telemedicine-based continuous NPPV is more cost-effective than traditional follow-up and care [18-22]. However, there are few economic assessments for patients with COPD, and their cost-effectiveness remains uncertain [23-27]. Therefore, more studies from different countries are needed to determine whether IoT-based telemonitoring is more cost-effective. Therefore, we built a Markov model based on a randomized controlled prospective study [28] to simulate and analyze the long-term cost-effectiveness of IoT-based management of home NPPV treatment in patients with COPD and hypercapnic chronic respiratory failure and provide a basis for decision-making regarding the rational selection of COPD treatment strategies.

## Methods

### Analytical Method

A Markov decision analytical model was built with Microsoft Excel 2016 software to estimate the long-term cost-effectiveness of IoT-based (intervention) versus standard NPPV (control) in patients with COPD and hypercapnic chronic respiratory failure.

### Patient Population

This study was based on a multicenter, prospective, randomized controlled trial (RCT; ChiCTR1800019536), which evaluated the effectiveness and safety of IoT-based management of home NPPV for patients with COPD and hypercapnic chronic respiratory failure in China. The trial was conducted over 12 months and involved 148 patients with a mean baseline age of 70.18 years and GOLD grades III-IV [29]. Patients were randomly allocated in a 1:1 ratio to the 2 study groups based on a computer-generated random number sequence implemented by an independent data center [28]. Both groups initiated oxygen therapy and NPPV treatment in the hospital, with an identical ventilator parameter titration protocol during hospitalization, and were discharged after meeting the treatment targets.

In the standard NPPV (control group; n=75), patients were advised to use home oxygen therapy for at least 12 hours per day and home NPPV intermittently for at least 8 hours per day, with continuous NPPV use during sleep, to maintain an oxygen saturation (SpO_2_) above the target of 88%.

In the IoT-based NPPV (intervention group; n=73), in addition to the home oxygen therapy for at least 12 hours per day and home NPPV for at least 8 hours per day, with continuous NPPV use during sleep, patients received additional IoT-based management for the full 12 months. The intervention group remotely collected ventilator data via a fourth-generation mobile communication technology (4G) system attached to the back of the ventilator and transmitted the data to the secured platform. The IoT cloud platform (Curative Medical Technology Inc) consists of a data collector, parse server, automatic processing server, data storage center, application interface server, and web server. The platform monitors clinical parameters such as use time, NPPV pressure, mask leaks, breaths per minute, and tidal volume information in real time, and automatically generates daily reports for medical staff. The platform delivers intervention suggestions when adverse reactions, leaks, or poor treatment efficacy occur. The IoT cloud platform triggers automatic alarms when the last 1-week average use is <5 h/night, mask leak is >60 L/min on 3 consecutive nights, or breaths are >25/min on 2 consecutive nights. Health care professionals respond promptly with telephone outreach; if necessary, emergency home visits are arranged, or patients are referred for in-hospital evaluation. Physicians adjust treatment plans based on platform data and patient feedback, with postdischarge management coordinated by family doctors, nurses, and health care providers (Air Liquide Healthcare). Health care professionals conduct in-home follow-ups at 1, 4, and 8 months after treatment, along with monthly telephone follow-ups, to provide health education, monitor the patient’s condition, and support long-term treatment adherence.

During the entire study duration, patients in both groups also received standard COPD treatment, including smoking cessation interventions and medication treatment, in accordance with international treatment guidelines.

Patients who were lost to follow-up, were noncompliant, or refused telemedicine, telephone contacts, or home visits were excluded from this analysis. A total of 125 patients (control group, n=67; intervention group, n=58) were included. Full trial details have been published elsewhere [28]. The baseline characteristics of the study population are shown in Table S1 in Multimedia Appendix 1. The groups appeared well balanced with no major differences in baseline characteristics.

### Ethical Considerations

The trial was approved by the medical ethics committee of Zhongshan Hospital, Fudan University (Shanghai, China; B2017-176R) and by local research and development committees at participating centers. In this trial, all patients provided written informed consent, and their data were anonymized. After enrollment, patients were provided with 1 year of free device use and transportation subsidies for 4 follow-up visits. Patients or the public were not involved in our research’s design, conduct, reporting, or dissemination plans.

### Markov Model Structure

**Figure 1.**
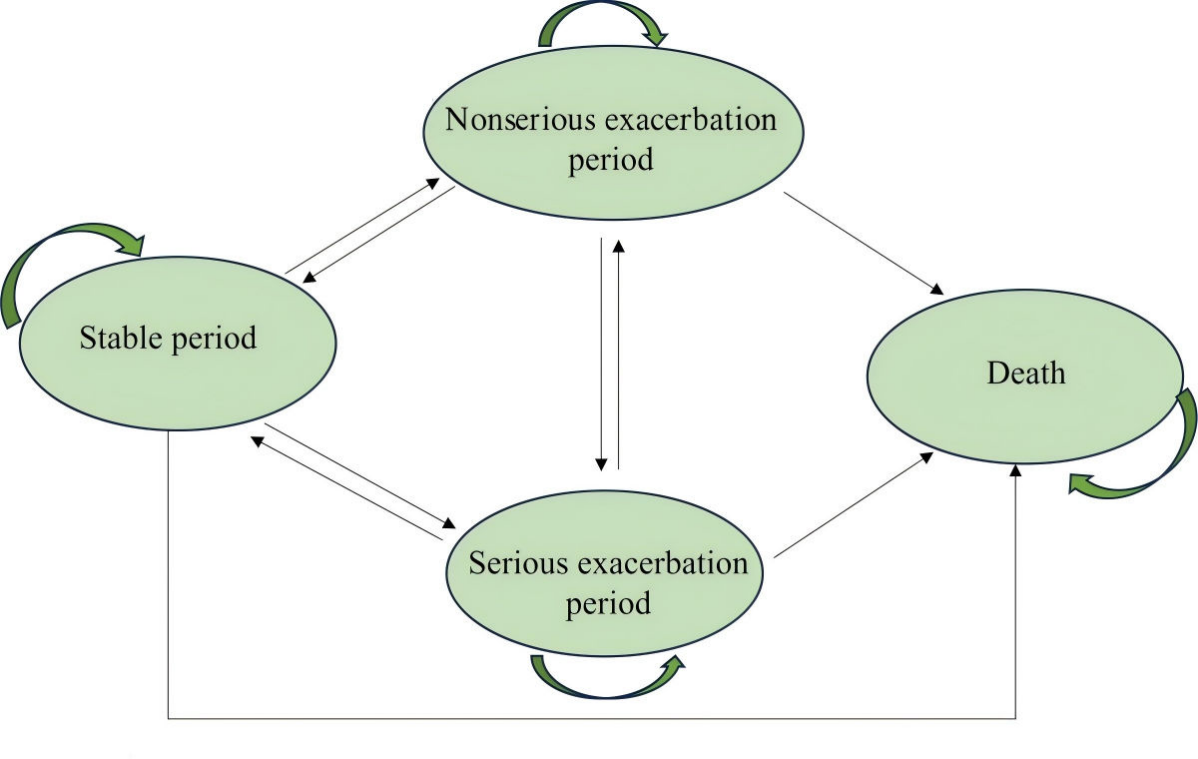
Markov model structure.

The model structure was adapted from Zhou et al [30] based on real-world COPD progression, consisting of 4 health states: stable period, nonserious exacerbation period, serious exacerbation period, and death (Figure 1). The stable period health state represents a period of stable health characterized by the absence of exacerbations. Exacerbation-related health states, namely nonserious exacerbation period or serious exacerbation period, were defined by the severity of an exacerbation and whether additional medical resources were needed. Nonserious exacerbations were events that led to patients needing medications adjusted (increasing the type of medication or dosage or changing medication), whereas serious exacerbations were defined as events that resulted in hospitalization. Patients could transition to the death health state from any other health state. Patients remained in one health state in each cycle and then transferred to another health state in the next cycle. The model adopted a lifetime horizon corresponding to 8 years, calculated based on the difference between China’s national average life expectancy in 2019 (77.3 years) and the average age of clinical trial patients included in this analysis (70.04 years). Each year represented a single model cycle, during which the 58 patients in the IoT-based management arm and the 67 patients in the standard management arm could progress throughout the model. The study adheres to Consolidated Health Economic Evaluation Reporting Standards (CHEERS) guidelines.

The RCT followed up with patients at 3, 6, and 12 months, so each patient had 3 periods of follow-up data, including COPD Assessment Test (CAT) scores, medication types, costs, and the number and costs of acute exacerbation hospitalizations. Due to the lack of patients’ baseline medication types, we could not identify whether patients had a medication adjustment in the first 3 months. Therefore, we could not identify whether patients were in the stable or nonserious exacerbation period in the first 3 months; we could only identify whether they were in the serious exacerbation period. The calculation of parameters in this study did not include data from this follow-up for patients whose health state could not be determined.

### Cost Parameters

Only direct costs collected as part of the trial, analyzed from the perspective of the Chinese health care payer, were included in this analysis. Cost components were grouped as either treatment costs, IoT costs, or hospitalization costs and were assumed to account for all aspects of care delivered during each health state (excluding death). Annual costs per patient differed for the IoT-based and standard management groups, as shown in Table 1. All costs are reported in Chinese Yuan (¥). Currency conversion to US dollars was based on the average exchange rate in 2019 (US $1=¥6.9).

**Table 1.** Cost parameters (¥ per patient per year).

Health state, groups, and items	Base value (SE)	Lower limit	Upper limit	Gamma distribution	
				α	β
Stable period^a^					
IoT^b^-based management					
Treatment	8913.18 (329.63)	8253.59	9572.78	731	12
Maintenance of telemedicine equipment	100.00 (—^c^)	90.00	110.00	—	—
Telephone contact	293.00 (—)	263.70	322.30	—	—
Health care professional home visits	2379.00 (—)	2141.10	2616.90	—	—
Standard management					
Treatment	8828.47 (324.00)	8192.63	9477.35	741	12
Nonserious exacerbation period^d^					
IoT-based management					
Treatment	10,792.54 (657.38)	9438.64	12,146.44	269	40
Maintenance of telemedicine equipment	100.00 (—)	90.00	110.00	—	—
Telephone contact	293.00 (—)	263.70	322.30	—	—
Health care professional home visits	2379.00 (—)	2141.10	2616.90	—	—
Standard management					
Treatment	10,157.65 (532.59)	9064.87	11,250.44	364	28
Serious exacerbation period^e^					
IoT-based management					
Hospitalization	29,675.74 (5456.09)	18,981.81	40,369.68	30	1003
Standard management					
Hospitalization	29,918.2 (3680.16)	22,705.09	37,131.32	66	453

a Stable period represents a period of stable health, characterized by the absence of exacerbations.

b IoT: Internet of Things.

c Not applicable.

d Nonserious exacerbation period represents events that lead to patients needing medications adjusted (increasing in type of medication or dosage or changing in medication).

e Serious exacerbation period defined as events that result in hospitalizations.

Treatment costs included device (oxygen concentrator and NPPV device) setup, maintenance, and support costs, considered one-time costs at the beginning of the treatment period based on standardized costs for the previous 12 months. Rental of ventilators and oxygen supply were also included, estimated based on the purchase price using the annuity method [31] over an assumed 3-year lifespan of the devices. Additionally, medication and follow-up examination costs in the stable and nonserious exacerbation period health states were identified based on patients’ self-reported case report forms and verified through electronic health records, where possible.

The next cost category, IoT costs, covered all costs associated with implementing IoT-based management and included telemedicine equipment maintenance, telephone contact, and health care professional home visits. The costs of telephone consultations and home visits from research and clinical teams were calculated based on hourly rates for different levels of health professionals.

Finally, hospitalization costs, covering all direct costs resulting from exacerbations, including medical and transportation costs, board and lodging, and nutrition and nursing, were collected again based on patient self-reported case report forms.

### Utilities

Quality-adjusted life years (QALYs) were used as effect indicators for this model. Utility values were derived from CAT scores using the following mapping equation (equation 1) [32]. This mapping was developed based on data from patients with COPD in Taiwan, using multiple linear regression with the ordinary least squares method, and was internally validated within the derivation sample, demonstrating good predictive performance.


(1) Utility=1.26848−0.02159×CAT−0.00188×age

In equation 1, *Utility* represents EQ-5D-3L health utility value, where 1 represents full health and 0 represents death, *CAT* represents CAT scores, and *age* represents patient age.

Utility values were calculated for each health state, and values used in this analysis are shown in Table 2, where a utility value of 1 represents perfect health, and a value of zero represents death.

**Table 2.** Utility parameters.

Health states	Base value (SE)	Lower limit	Upper limit	α^a^	β^a^
Stable period	0.6195 (0.0121)	0.5956	0.6434	997	612
Nonserious exacerbation period	0.5960 (0.0181)	0.5598	0.6323	437	296
Serious exacerbation period	0.5319 (0.0127)	0.5065	0.5573	820	722
Death	0 (0)	—^b^	—	—	—

a The α and β values represent the shape parameters of the beta distribution.

b Not applicable.

### Transition Probabilities

Transition probabilities were primarily derived from the clinical trial and supplemented by official Chinese databases. The annual natural mortality rate of the population was obtained from the *Chinese and Employment Statistics Yearbook 2021 “National Death Population States by Age and Sex in 2020 (November 1, 2019 to October 31, 2020)”* [33] and converted into the natural mortality rate of participants aged 40 to 80 years of whom 84% were male in the clinical trial. This was used to calculate the transition probabilities of patients from the stable period and nonserious exacerbation period health states to death. Other transition probabilities were calculated based on the patient population in the clinical trial. The conversion from incidence to probability was obtained by using the formula (equation 2) [34]. All model transition probabilities are shown in Table 3.

**Table 3.** Annual transition probabilities.

Health state transition	IoT^a^-based management			Standard management			References
	Base value (SE)	α^b^	β^b^	Base value (SE)	α	β	
Stable period							
Stable period	0.4760 (—^c^)	—	—	0.4332 (—)	—	—	—
Nonserious exacerbation	0.3324 (0.0712)	14	29	0.1993 (0.0663)	7	35	[28]
Serious exacerbation	0.1829 (0.0680)	6	31	0.3588 (0.0672)	18	32	[28]
Death	0.0087^d^ (—)	—	—	0.0087 (—)	—	—	[33]
Nonserious exacerbation							
Stable period	0.4134 (0.1212)	6	16	0.3588 (0.1041)	7	13	[28]
Nonserious exacerbation	0.3438 (—)	—	—	0.2191 (—)	—	—	—
Serious exacerbation	0.2341 (0.1292)	2	10	0.4134 (0.0989)	10	14	[28]
Death	0.0087 (—)	—	—	0.0087 (—)	—	—	[33]
Serious exacerbation							
Stable period	0.2835 (0.0844)	8	20	0.2835 (0.0844)	8	20	[28]
Nonserious exacerbation	0.2425 (0.0837)	6	19	0.2425 (0.0837)	6	19	[28]
Serious exacerbation	0.3261 (—)	—	—	0.3261 (—)	—	—	—
Death	0.1479 (0.0625)	5	27	0.1479 (0.0625)	5	27	[28]

a IoT: Internet of Things.

b The α and β values represent the shape parameters of the beta distribution.

c Not applicable.

d 0.0087 represents the annual natural mortality rate.


(2) $$tp_{t}=1-exp(-r*t)$$

In Equation 2, *tp*_*t*_ represents the probability of event occurrence during *t*, *r* means the incidence rate during *t*, and *t* represents the study time.

Details of the cost-effectiveness analysis can be found in the Multimedia Appendix 1.

### Outcomes

In line with the *Chinese Guidelines for Pharmacoeconomic Evaluation (2020*) [35], future costs and QALYs were discounted at 5% each year. Model outputs included total per-patient costs and QALYs and an incremental cost-effectiveness ratio (ICER), calculated to express the incremental difference in costs and QALYs between IoT-based management and standard management groups. The ICER was judged against a willingness-to-pay (WTP) threshold equal to per capita gross domestic product (GDP). The 2019 per capita GDP was ¥70,100 in China, according to the official data from the National Bureau of Statistics. IoT-based management was considered cost-effective when the ICER value was lower than ¥70,100 per QALY.

### Sensitivity Analysis

#### One-Way Sensitivity Analysis

A one-way sensitivity analysis (OWSA) was used in the study to understand how changes in key parameters influenced results. The transition probabilities were not included in the OWSA because they were correlated with each other. All inputs were adjusted by the 95% CIs of the base value, with the exception of the discount rate, which was varied from 0% to 8%, and the cost of IoT, which was adjusted by −10% to +10% of the base value. Results of the OWSA are presented in a tornado chart.

#### Probabilistic Sensitivity Analysis

A probabilistic sensitivity analysis (PSA), using Monte Carlo simulations with 1000 iterations, was also performed to incorporate parameter uncertainty into the model analysis. In each iteration, parameter values were randomly drawn from specified distributions and results were presented on a scatter plot. A cost-effectiveness acceptability curve was also used to show the probability of IoT-based management being cost-effective against standard management given different WTP thresholds.

## Results

### Baseline Results

In the base-case analysis, total per-patient costs were ¥94,632.50 for the IoT-based management group and ¥91,025.24 for the standard management group; total QALYs were 3.49 and 3.25, respectively, across the model time horizon. Compared with the standard management group, IoT-based management increased costs by ¥3607.26 but also increased QALYs by 0.24 per patient across the time horizon, resulting in an ICER of ¥15,030.25 per QALY. As the ICER was substantially less than the WTP threshold of ¥70,100 per QALY, IoT-based management was cost-effective compared with standard management. These results are presented in Table 4.

**Table 4.** Base case results.

Group	Total costs (¥)	Total effectiveness (QALYs^a^)	Incremental cost (¥)	Incremental effectiveness (QALYs)	ICER^b^ (¥/QALY)
IoT^c^-based management	94,632.50	3.49	—^d^	—	—
Standard management	91,025.24	3.25	3607.26	0.24	15,030.25

a QALY: quality-adjusted life year.

b ICER: incremental cost-effectiveness ratio.

c IoT: Internet of Things.

d Not applicable.

### One-Way Sensitivity Analysis

**Figure 2.**
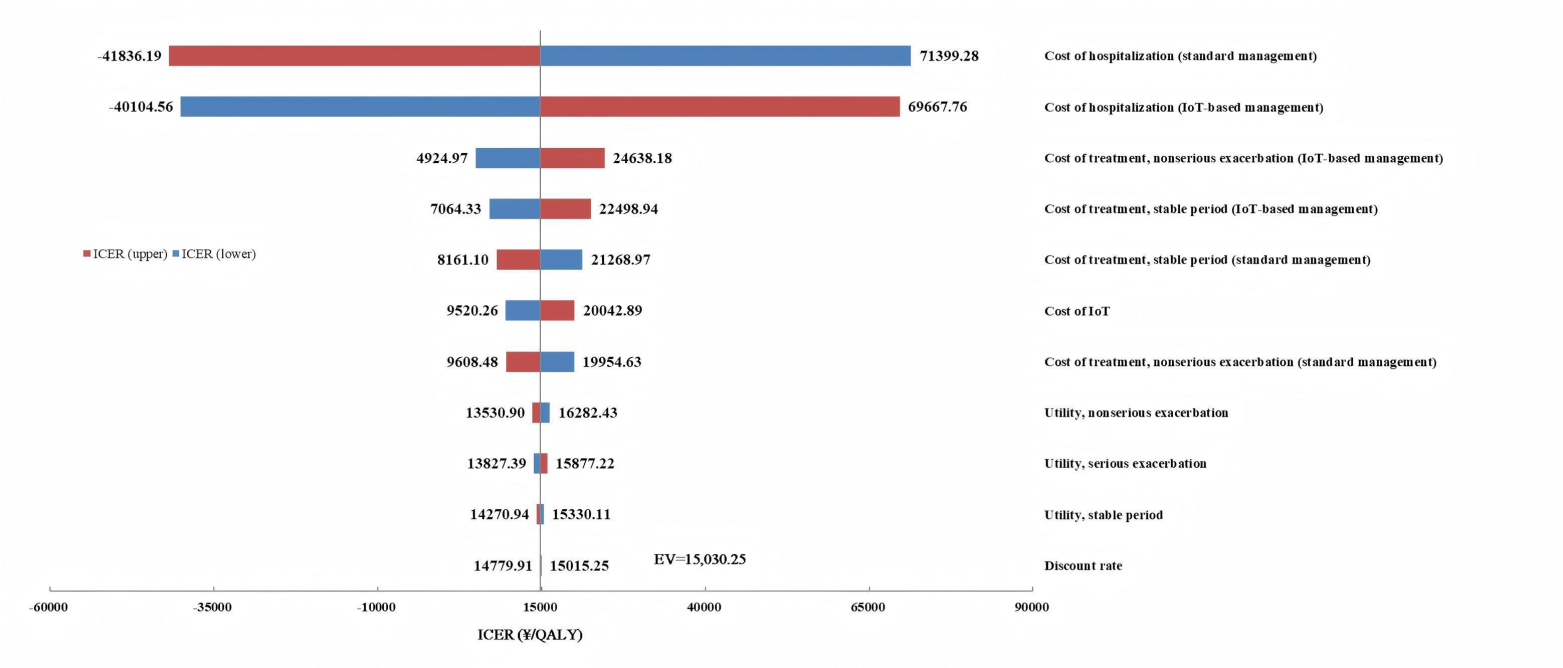
One-way sensitivity analysis tornado chart. EV: expected value; ICER: incremental cost-effectiveness ratio; IoT: Internet of Things; QALY: quality-adjusted life year.

Results of the OWSA, displayed in Figure 2, showed that the parameter that drove the greatest change in ICER results was the hospitalization cost of a serious exacerbation for the standard management group. The second most influential parameter was the hospitalization cost of a serious exacerbation for the IoT-based management group. All other parameter changes had comparatively less influence on model results.

### Probabilistic Sensitivity Analysis

**Figure 3.**
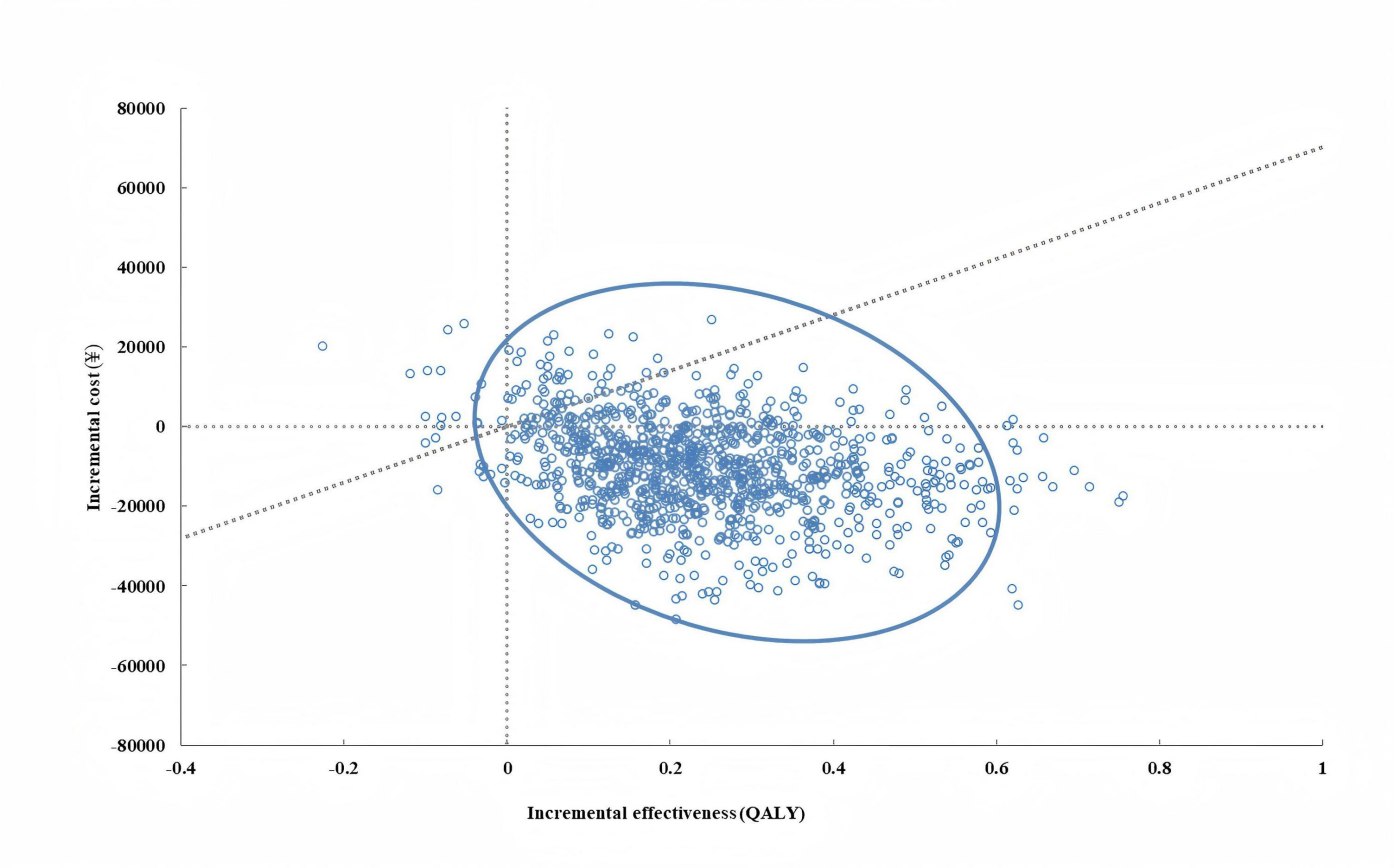
Monte Carlo simulation scatterplot. Dotted line indicates willingness to pay=¥70,100 per quality-adjusted life year (QALY).

**Figure 4.**
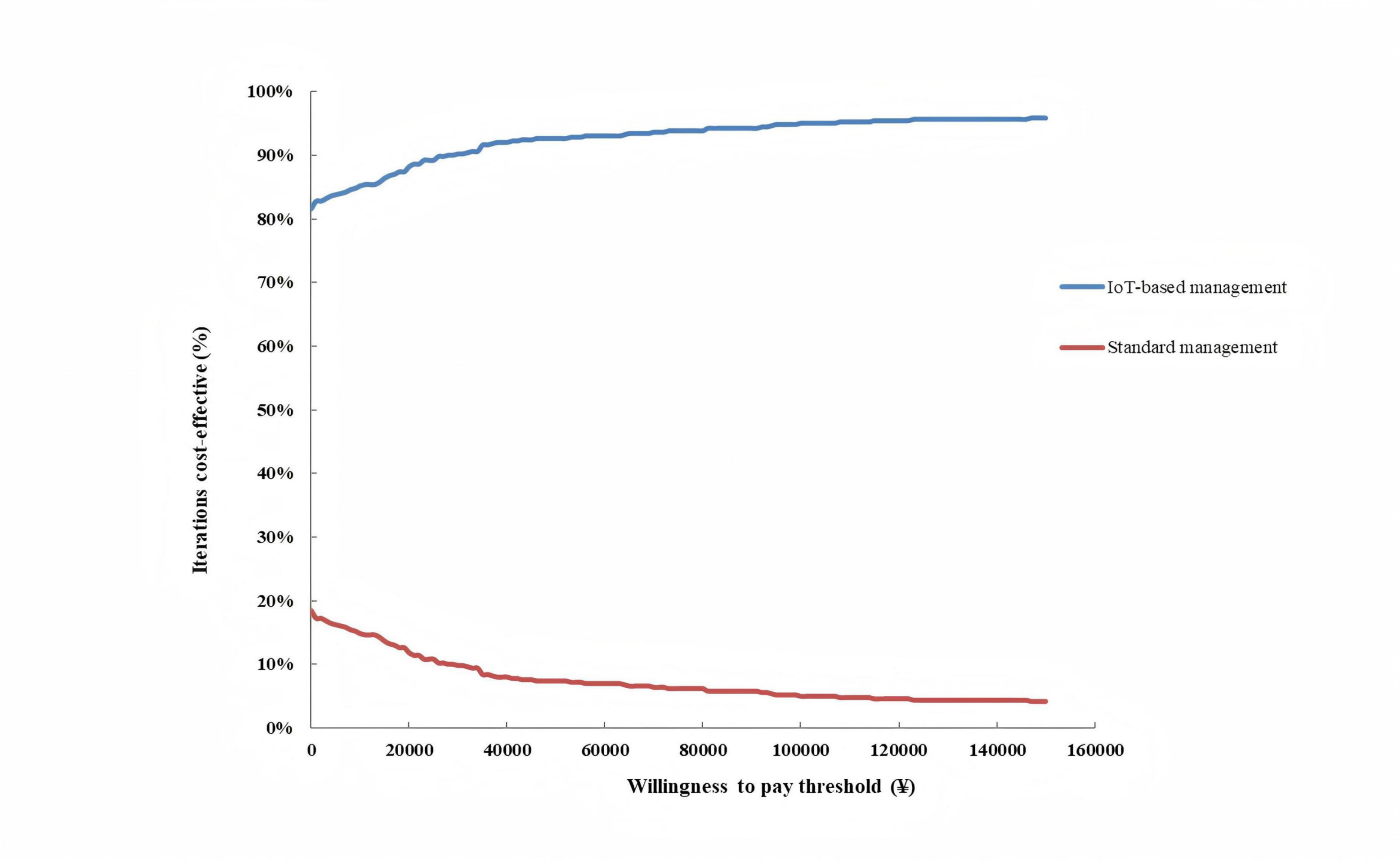
Cost-effectiveness acceptability curve. IoT: Internet of Things.

Results of the PSA, presented in Figure 3, showed the uncertainty around the model’s cost and QALY estimates. A total of 93.6% of iterations fell below the WTP threshold (indicated by the dotted line), indicating that IoT-based management had a 93.6% probability of being cost-effective relative to standard management at the given WTP threshold. This relationship between the probability of being cost-effective as the WTP threshold changes is shown in Figure 4. Additionally, in 80.2% of PSA iterations, IoT-based NPPV was more effective (accumulating more QALYs) and cheaper than standard NPPV.

## Discussion

This study demonstrated that, compared with standard NPPV, IoT-based NPPV was cost-effective for patients with COPD and hypercapnic chronic respiratory failure in China. The base case analysis achieved an ICER of ¥15,030.25 per QALY, which was substantially below the ¥70,100 per QALY WTP threshold. PSA results provided robust evidence further supporting the cost-effectiveness of IoT-based NPPV. They confirmed a high probability (93.6%) of IoT-based NPPV being cost-effective, as well as a high probability (80.2%) of IoT-based NPPV being dominant (ie, cheaper and more effective).

Despite widespread understanding of the significant clinical and economic burdens imposed by COPD, this is, to the best of our knowledge, the first economic evaluation of IoT-based home NPPV treatment for patients with COPD in China. The level of existing economic evidence on the topic is limited, and results differ across countries. Meanwhile, a systematic review [36] highlighted the poor quality of this existing evidence. Five studies included in the systematic review were evaluated as low quality, and only one was evaluated as moderate quality. Further, none of the 6 studies included in the analysis were conducted in China. Consistent with our findings, an Italian study [23] found that a nurse-centered tele-assistance prevented hospitalizations and was a cost-effective intervention for patients with chronic respiratory failure receiving oxygen or home mechanical ventilation. A Spanish study [24] also found that a telehealth program was cost-effective for patients with severe COPD without comorbidities. However, telehealth was not cost-effective compared with usual care in the base case. A Dutch study showed that home initiation of chronic noninvasive ventilation in stable hypercapnic patients with COPD, with the use of telemedicine, is noninferior to in-hospital initiation, is safe, and reduces costs by more than 50% [19]. A study in the United Kingdom also concluded that telemonitoring in patients with COPD was not cost-effective. A possible rationale for the United Kingdom study not being cost-effective is that general practitioner home consultations were more frequent, with higher general practitioner costs in the telemonitoring arm, but differences in QALYs over the trial period were not significant between trial arms [25]. More specifically, existing economic evidence supported the use of telemonitoring in managing COPD [21,37].

The medical IoT is a key part of the digital transformation of health care, as it creates new health care delivery models and enables stakeholders to rebuild the workflows, contributing to increased productivity, enhanced patient experience, and improved effectiveness of home management for chronic diseases [38,39]. Specifically, IoT enables remote patient management and health education through architectures such as real-time monitoring via sensors, cloud-based data processing, and clinical feedback loops, thereby improving patient adherence to home-based therapy. At the same time, it relies on stringent data encryption and privacy protection mechanisms to ensure the long-term safety of patients.

A key outcome from the multicenter, prospective RCT, which formed the basis of this economic evaluation, was that compared with NPPV alone, the addition of an IoT treatment program reduced acute COPD exacerbation-related readmission frequency during the study period. The acute COPD exacerbation-related readmission frequency during the study period was 0.57 (95% CI 0.34‐0.80) for the IoT-based NPPV group and 0.94 (95% CI 0.68‐1.21) for the standard NPPV group (*P*=.05) [28]. The possible reason is that patients with IoT-based management are better able to manage their own condition, signs of exacerbations are identified earlier than without IoT management, and patients' adherence is improved [28]. The reduction in hospitalization costs for the IoT-based NPPV group partially offset the investment in IoT services. Reducing these admissions also reduced organizational burden and potentially lessened pressure on the Chinese health care system.

There exists a disproportionately high burden of global morbidity and mortality caused by chronic respiratory diseases in low- and middle-income countries (LMICs) [40], whereas LMICs typically face the challenge of scarce health care resources, such as inadequate coverage of health care facilities, a shortage of human resources for health, and poor quality of health services. The cost-effectiveness results of this study showed that IoT-based NPPV management could produce greater health and economic outcomes for a given budget. Such interventions are likely to have a greater impact in LMICs, contributing to increased efficiency in allocating limited health resources and increasing the accessibility of health services in LMICs, as well as reducing the pressure on the operation of the health system and the economic burden of COPD-related diseases. Therefore, this intervention should be considered a priority in LMICs. Nevertheless, the scalability of such interventions may depend on local digital infrastructure, workforce readiness, and regulatory frameworks, which vary substantially across LMICs [41]. Therefore, policymakers should consider phased implementation, investment in digital capacity building, and the development of data governance standards to ensure safe and sustainable expansion of IoT-enabled care. Particular attention should also be given to the accessibility of IoT-based management for vulnerable populations, such as those in low-income groups, to avoid exacerbating digital health inequalities.

This study contributes a new perspective to existing international research on the cost-effectiveness of IoT-based home NPPV for patients with COPD, indicating that IoT-based NPPV is cost-effective for patients with COPD and hypercapnic chronic respiratory failure in China. However, this study has several limitations. First, this economic evaluation was based primarily on the results of a single multicenter, prospective RCT, and this restricted clinical trial sample size may differ in some characteristics from the true population of the Chinese health care payer. This approach also means that trial limitations may also impact the inference of model results. Second, given that no studies on utility value measurement for acute exacerbation or stable patients with COPD in mainland China exist, the utility values for patients with COPD adopted in this analysis were derived from a mapping formula based on a study in Taiwan. Given the possible differences in patient characteristics, the formula may have underestimated the utility values of patients with severe disease. Finally, transitions to the nonserious exacerbation health state were determined based on the medication adjustments, as opposed to an objective diagnosis from a doctor, and this may introduce certain bias to the flow of patients throughout the model.

In conclusion, this study demonstrated that from a Chinese health care payer perspective, IoT-based NPPV was cost-effective compared with standard NPPV for patients with COPD and hypercapnic chronic respiratory failure. Further large-sample RCTs, additional real-world data, and more widespread implementation of IoT-based NPPV throughout China should be sought to validate this.

## Supplementary material

10.2196/71340 Multimedia Appendix 1 Baseline characteristics and supplementary methods for health-state classification, utility and cost estimation, transition probability calculation, and probabilistic sensitivity analyses.

## Abbreviations

CAT: COPD Assessment Test
CHEERS: Consolidated Health Economic Evaluation Reporting Standards
COPD: chronic obstructive pulmonary disease
GDP: gross domestic product
ICER: incremental cost-effectiveness ratio
IoT: Internet of Things
LMIC: low- and middle-income country
NPPV: noninvasive positive pressure ventilation
OWSA: one-way sensitivity analysis
PSA: probabilistic sensitivity analysis
QALY: quality-adjusted life year
RCT: randomized controlled trial
WTP: willingness-to-pay
