# Supplementary material for: Cost-Effectiveness of Internet of Things–Based Management of Home Noninvasive Positive Pressure Ventilation in Patients With Chronic Obstructive Pulmonary Disease and Hypercapnic Chronic Respiratory Failure: Trial-Based Economic Evaluation
Source: JMIR Mhealth Uhealth. 2026 May 14;14:e71340. doi: 10.2196/71340 (PMC13175562; doi:10.2196/71340)
Supplement: Multimedia Appendix 1 [file mhealth-v14-e71340-s001.docx]

**SUPPLEMENTARY APPENDIX**

**Cost-Effectiveness of Internet of Things-–Based Management of Home Noninvasive Positive Pressure Ventilation in Patients With Chronic Obstructive Pulmonary Disease and Hypercapnic Chronic Respiratory Failure: Trial -Based Economic Evaluation**

**Baseline characteristics**

**Supplementary Table S1. Baseline characteristics**

| Characteristic | Total  N=125^1^ | NPPV alone  N = 67^1^ | NPPV plus IOT  N = 58^1^ | *p*-value^2^ |
| --- | --- | --- | --- | --- |
| Age | 70.04 ± 6.62 | 70.10 ± 6.97 | 69.97 ± 6.26 | 0.911 |
| Sex |  |  |  | 0.914 |
| Female | 20 (16.00%) | 10 (14.93%) | 10 (17.24%) |  |
| Male | 105 (84.00%) | 57 (85.07%) | 48 (82.76%) |  |
| BMI, kg/m^2^ | 22.25 ± 4.63 | 21.85 ± 4.64 | 22.71 ± 4.62 | 0.302 |
| Former smoker |  |  |  | 0.543 |
| No | 22 (17.60%) | 10 (14.93%) | 12 (20.69%) |  |
| Yes | 103 (82.40%) | 57 (85.07%) | 46 (79.31%) |  |
| Hypertension |  |  |  | 0.330 |
| No | 56 (45.16%) | 33 (50.00%) | 23 (39.66%) |  |
| Yes | 68 (54.84%) | 33 (50.00%) | 35 (60.34%) |  |
| Diabetes |  |  |  | 0.488 |
| No | 105 (84.68%) | 54 (81.82%) | 51 (87.93%) |  |
| Yes | 19 (15.32%) | 12 (18.18%) | 7 (12.07%) |  |
| CHD |  |  |  | >0.999 |
| No | 100 (80.65%) | 53 (80.30%) | 47 (81.03%) |  |
| Yes | 24 (19.35%) | 13 (19.70%) | 11 (18.97%) |  |
| OSA |  |  |  | 0.859 |
| No | 116 (93.55%) | 61 (92.42%) | 55 (94.83%) |  |
| Yes | 8 (6.45%) | 5 (7.58%) | 3 (5.17%) |  |
| FEV_1_, L | 0.68 ± 0.30 | 0.71 ± 0.37 | 0.64 ± 0.20 | 0.242 |
| FEV_1_ predicted | 26.56 ± 9.05 | 26.78 ± 9.87 | 26.31 ± 8.09 | 0.771 |
| FVC, L | 1.49 ± 0.55 | 1.54 ± 0.60 | 1.43 ± 0.49 | 0.274 |
| FEV_1_/FVC (%) | 46.85 ± 11.06 | 46.67 ± 11.46 | 47.05 ± 10.68 | 0.849 |
| PaCO_2_, mmHg | 66.00 ± 12.04 | 66.48 ± 13.55 | 65.43 ± 10.11 | 0.621 |
| HCO_3_, mmol.L | 37.65 ± 6.01 | 37.39 ± 6.12 | 37.94 ± 5.92 | 0.609 |
| pH | 7.38 ± 0.05 | 7.37 ± 0.06 | 7.38 ± 0.05 | 0.509 |
| Pulmonary hypertension |  |  |  | 0.495 |
| No | 56 (45.90%) | 27 (42.19%) | 29 (50.00%) |  |
| Yes | 66 (54.10%) | 37 (57.81%) | 29 (50.00%) |  |
| SRI score | 128.60 ± 25.09 | 128.21 ± 24.12 | 129.05 ± 26.37 | 0.855 |
| mMRC score | 3.10 ± 0.82 | 3.06 ± 0.82 | 3.14 ± 0.83 | 0.603 |
| CAT score | 28.52 ± 6.79 | 28.68 ± 7.17 | 28.33 ± 6.39 | 0.772 |
| IPAP, cm H_2_O | 16.08 ± 3.00 | 15.65 ± 3.04 | 16.57 ± 2.90 | 0.089 |
| EPAP, cm H_2_O | 4.63 ± 0.77 | 4.57 ± 0.87 | 4.69 ± 0.65 | 0.377 |
| ^1^Mean ± SD; n (%) | | | | |
| ^2^Welch Two Sample t-test; Pearson's Chi-squared test | | | | |

**Calculation procedure**

The RCT followed up patients at the 3-month, 6-month, and 12-month, so each patient had 3 periods of follow-up data, including CAT scores, medication types and costs, number and costs of acute exacerbation hospitalizations. Based on the medication use and serious exacerbation hospitalizations times at each follow-up visit, patients were judged to be in a health state at each follow-up period. Patients who had medications adjusted (increase in type of medication, increase in dosage, change in medication) compared to the previous follow-up visit were in Non-serious exacerbation period at the current follow-up period, those who had serious exacerbation hospitalizations were in Serious exacerbation period at this follow-up period, and those who had both medications adjusted and hospitalization were judged to be in Serious exacerbation period, and patients who experienced neither were in the Stable period. Due to the lack of patients' baseline medication types, we could not identify whether patients had a medication adjustment in the first 3 months. Therefore, we could not identify whether patients were in Stable or Non-serious exacerbation period in the first 3 months, can only identify whether they were in Serious exacerbation period. The calculation of parameters in this study didn’t include data from this follow-up for patients whose health state was not judged.

**1. Utility calculation**

Utility values were only related to patients’ health states, without distinguishing between the intervention and control groups. The utility values for the same health state in the three follow-up periods were averaged to calculate the health utility value for each health state. For example, the utility value for Serious exacerbation period was calculated by averaging the utility values of patients in the Serious exacerbation period at 3-month, 6-month, 12-month follow-up visit. Calculated data for Stable period and Non-serious exacerbation period were for 6-month, 12-month follow-up visit.

**2. Cost calculation**

(1)Treatment costs: Calculate the monthly treatment costs for each follow-up period separately, multiply 1 year to get annual treatment costs, and then average the annual treatment costs for three follow-up periods in the same health state to get the annual treatment costs per patient for each health state. Annual treatment costs per patient in Stable period were calculated by averaging the monthly treatment costs at 6-month, 12-month follow-up visit in Stable period. The calculation method for the Non-serious exacerbation period was the same as that for the Stable period. (2)Hospitalization costs: The hospitalization expenses of each acute exacerbation were counted to calculated average hospitalization expenses of each acute exacerbation, and the average annual hospitalization numbers for patients with acute exacerbation were calculated. Annual hospitalization costs per patient were obtained by multiplying average annual hospitalization numbers and average hospitalization expenses of each acute exacerbation.

Data on patients who died during follow-up period were not included in the calculation of costs and utility values.

**3. Transition probability**

The patients’ health state of 4-6 month was used as the start health state and 7-12 month as the transferring health state, with a follow-up time of 9 months. The probability of transition from Stable, Non-serious exacerbation and Serious exacerbation to else health states (excluding self-circulation and death) was calculated for that follow-up time and converted to a one-year transition probability by the formula. For example: there were A patients in Stable period at 4-6 months, B of A patients were in Non-serious exacerbation period at 7-12 months. Transition probability (from Stable period to Non-serious exacerbation period in 1 year) =1-exp(-B/A*12/9).The transition probabilities for the Stable and Non-serious exacerbation period to death were obtained from the annual natural mortality rate from the statistical yearbook, normalized by the age and sex ratio of the RCT population. Transition probability from Serious exacerbation period to death: the patients’ health state of 1-3 month was used as the start health state and 1-12 month as the transferring health state, with a follow-up time of 1 year. For example: there were C patients in Serious exacerbation period at 1-3 months, D of C patients were death in hospital at 1-12 months. Transition probability (from Serious exacerbation period to Death in 1 year) =1-exp(-D/C). Patients who were hospitalized and died at one follow-up visit were judged to be in Serious exacerbation period when determining the transfer of health state.

The transition probabilities of self-circulation were obtained by subtracting 1 from the other transition probabilities. Because there were both transferred out and transferred in the self-loop, which were difficult to calculate.

**4. Probabilistic sensitivity analyses**

Distribution parameters were calculated by mean and standard error.
